# Development of a New Intelligent Joystick for People with Reduced Mobility

**DOI:** 10.1155/2018/2063628

**Published:** 2018-03-22

**Authors:** Makrem Mrabet, Yassine Rabhi, Farhat Fnaiech

**Affiliations:** The National Higher School of Engineering of Tunis (ENSIT), Laboratory of Signal Image and Energy Mastery, LR13ES03 (SIME), University of Tunis, Tunis, Tunisia

## Abstract

Despite the diversity of electric wheelchairs, many people with physical limitations and seniors have difficulty using their standard joystick. As a result, they cannot meet their needs or ensure safe travel. Recent assistive technologies can help to give them autonomy and independence. This work deals with the real-time implementation of an artificial intelligence device to overcome these problems. Following a review of the literature from previous work, we present the methodology and process for implementing our intelligent control system on an electric wheelchair. The system is based on a neural algorithm that overcomes problems with standard joystick maneuvers such as the inability to move correctly in one direction. However, this implies the need for an appropriate methodology to map the position of the joystick handle. Experiments on a real wheelchair are carried out with real patients of the Mohamed Kassab National Institute Orthopedic, Physical and Functional Rehabilitation Hospital of Tunis. The proposed intelligent system gives good results compared to the use of a standard joystick.

## 1. Introduction

The intelligent methodologies, such as artificial neural network, genetic algorithms, and fuzzy logic, stand as a major base in processing high-level inferences in order to control systems and to deal with nonlinear and complex problems. This technique based on human reasoning plays a pivotal role in many fields such as medicine, robotics, and engineering. Indeed, it is widely deployed in the development of healthcare smart technologies like electric wheelchairs (EWC), where many intelligent prototypes have been designed to fit with every user's exigency.

These users have difficulty driving their standard electric wheelchairs; they are either elderly, disabled, or severely impaired. In fact, a clinical survey [1] stated that

- 10% of patients surveyed cannot use the standard electric wheelchair in their daily activities.
- 40% of regular users of electric wheelchairs find it difficult to manage tasks such as going through open doors, and almost 9% find this impossible without assistance.
- 18% to 26% of patients cannot use a manual or motorized wheelchair because they lack the required motor skills, strength, or visual acuity.

In order to overcome these deficiencies and hence design new EWC using advanced technologies to reach high performances and secure systems, an important number of researchers aim to develop new technologies and functionalities to solve this problem. In [2], the authors use the voice recognition to move the EWC. In fact, based on a control database, the speech controller identifies the voice and applies the command. Another method used in the literature [3] consists of fusing an eye tracker with an infrared technology (IR), the user stares at the desired direction then the IR sensors send eye location to the controller. The head gesture is also an innovative method of controlling an EWC, using cameras, body sensors, or a facial recognition system to detect movement of the user's head [4]. In [5], the authors use a tube connected to the control system on which the user blows or sucks. The control depends on the amount of air and the hardness or softness of the sips and puffs. The brain-computer interface (BCI) is a direct communication between the brain and the computer, where a set of electrodes attached to the scalp collects brain signals and transfers them to a computer to be pretreated. It is used in [4], to control the EWC.

Despite these highly developed technologies, the joystick remains the most widely used input device and others are little marketed according to clinical research [1], more than 80% of EWC users operate their wheelchairs with the joystick, 9% use a head or chin control interface, and only 10% use it for all other types of control. All the interfaces mentioned above have predetermined commands to move the wheelchair. It allows only four different commands (forward, backward, left, and right) at a predefined speed. In addition, these control types have a tedious impact on the patients when they drive for a long duration.

Furthermore, electric wheelchairs are now becoming smarter with the implementation of intelligent algorithms that assist in the driving process.

Therefore, in this work, a new method of controlling EWC is proposed based on the use of the classic joystick to which we have added an artificial intelligence algorithm to correct all the disabled movements of the patient's hand.

## 2. Problem Description

Hand-operating control difficulties are the main reasons that create a huge lack of security while steering an electric wheelchair.

Firstly, in the case of muscle tone, poor endurance, or decreased strength, a wheelchair user is in a position of evident weakness, where its ability to propel the wheelchair is extremely deteriorated. Moreover, when the place or the navigation room is crowded or the floor is not perfectly smooth and flat, crashing and falling become almost certain to result, which makes the patient more likely to make accidents and more severely injured, due to the slow-moving hand response when he faces obstacles.

In order to ensure his security, he should quickly sense and react to each situation; he should correct the undesirable hand movement.

The bad control or falling down from a WC or crashing things will cause many physical and psychological problems for old and handicapped persons.

## 3. Related Work

Conventional wheelchairs consist of a joystick to perform numerous control tasks. The user needs to be sufficiently flexible to reach and operate them. Some patients are unable to manipulate the joystick of the wheelchair with their arms due to a lack of strength or problems in the superior members caused by Parkinson and quadriplegics diseases.

Many works based on wheelchairs have been proposed to improve its usability [6, 7]. Human interface for easy operation of the intelligent wheelchair is the most popular research issue. The joystick mapping study is also an investigation issue to control the wheelchairs. Rabhi et al. [8] describe an intuitive human interface by changing the standard joystick with another visual interface and also the previous method of mapping the joystick. This method requires the addition of other equipment to the wheelchair, such as a camera and additional treatment to detect the patient's hand.

Many standard joysticks contain low-pass filters. In [9], the authors have built-in damp features to suit the nature of the rubber boot around the joystick. However, low-pass filters are adequate to filter out some unintended movements such as tremor. Vishnu et al. present in [10] an algorithm which employs natural image features for indoor corridor navigation. This algorithm is then fused with standard joystick input from the user for progressive assistance and trajectory correction. In addition, other wheelchair-based works have been proposed to improve its ergonomics. The human interface for easy use of the electric wheelchair is the most popular research topic. In [11], the author proposes a new method of classifying human facial movement based on multichannel frontal biosignals. Another hands-free control system based on visual recognition of head movements is developed in [12]. The author in [13] developed a voice-operated electric wheelchair. The user can control the wheelchair by voice commands. Nevertheless, many types of interfaces need to be developed to allow for more variety in the wheelchair, thus reducing fatigue/stress, minimizing downtime due to adaptation, and maximizing the effective use of the control module. Unfortunately, most of these interfaces have predetermined controls to move the wheelchair. They have a major defect that requires additional hardware to collect these signals. In addition, it allows only four different commands (front, rear, left, or right) at a preset speed.

## 4. Proposed Solution

In this work, persons with severe upper extremity impairments are considered. These people are unable to maintain their wheelchairs' joystick conveniently and to precise their navigation path to move toward the desired point with the suitable acceleration. To solve these limitations, we integrate into the electric wheelchair a behavior control system that shares the control with the user and ensures a safe navigation with a real-time support. This control system is based on an artificial intelligence method. Therefore, we use the recurrent neural network algorithm to design an intelligent controller that constantly corrects undesirable movements of the patient's hand and ensures smooth and safe navigation, respectively. Recurring neural networks (RNNs) have no limitations and are very efficient in signal sequence modeling. They are very useful for modeling the movements of the hands [14, 15].

This study is aimed at people with severe disabilities of the upper limbs; we offer them an intelligent system integrated into the standard wheelchair joystick allowing to move to the desired point with an appropriate acceleration. The system uses an intelligent algorithm to control and assist variable speed navigation (such as standard joystick navigation). This proposed control system does not require sensors or devices attached to the user's body or a special camera on the wheelchair.

**Figure 1:**
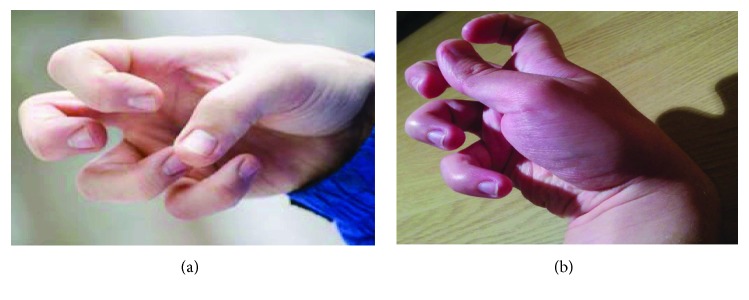
The posture of a dystonia patient's hand.

For our target users, this modality seems very appropriate: this new intelligent joystick can be manipulated even with a tight hand posture, Figure 1. In addition, using the proposed smart interface requires less muscular effort than a standard joystick.

In this section, a global presentation of the system is proposed, it explains the used strategy and presents the proposed solution.

## 5. Materials and Methods

**Figure 2:**
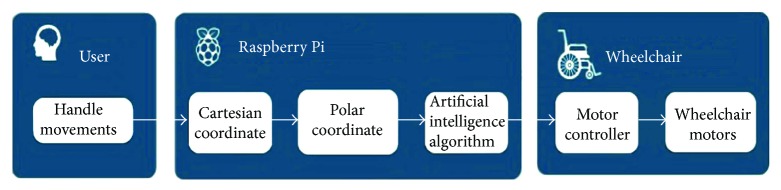
The proposed system structure.

Figure 2 shows the overall architecture of the intelligent assistance system and the connection between its elements. In the next step, the use of each element of the system is explained in detail.

The aim of the project is to develop an intelligent platform that is easily adaptable to any electric wheelchair and to help many people with physical limitations and seniors who have difficulty maneuvering their joysticks. A real prototype was created by adopting a conventional electric wheelchair that has already been used in other works in our laboratory, such as in [8]. The intelligent control system consists of two devices, which are the standard joystick and Raspberry pi2. In order to safely simulate algorithms and methodologies, a 3D simulator was also used. This simulator makes it possible to create a virtual world in which the user can drive an electric wheelchair whose behavior is similar to that of the real prototype with real parameters of the material presented in [8]. The virtual world was developed using the Unity 3D engine, used for 3D game modelling. It provides a toolkit for creating a 3D simulator and for many video games. Wheelchair control mapping is very important to move in the right direction. However, unfortunately, some people did not have the ability to use the standard controller as we have presented previously. That is why different ways of mapping the joystick control were tested and in this work, we have proposed an algorithm based on the recurrent neural network to make the necessary corrections of this mapping.

The joystick controller is the tool used to manoeuvre an EWC with a high degree of flexibility; it is easily adjusted to suit the driving needs of the individual and to displace independently without the assistance of another person. It converts the hand movement into mathematical symbols. Indeed, when the user begins propelling his WC, the joystick receives Cartesian coordinates (*x* and *y*) and converts them into polar coordinates (distance and angle). The received value for the *x*-axis increases as the stick moves to the right, and the value of the *y*-axis gets greater as the stick moves away from the user. After the control process, it reconverts again the outputs into an analog voltage. Our EWC is equipped with a new VSI controller, which is a multimodule control system.

The joystick mapping can adjust the suitable response behaviour of the electric wheelchair to the patient's control. The joystick controller converts the hand movement into mathematical symbols. Indeed, when the user begins propelling his wheelchair, the joystick receives the coordinates and converts them into signals. The joystick handle movement is represented in a Cartesian coordinate system, with two axes, *x* and *y*, where the *x*-axis and *y*-axis are the rotational and translational command simulation of the joystick.

In this work, a control command of (1) equates to maximal forward or left rotation and a control command of (−1) equates to maximal reverse or right rotation by the wheelchair. A command of (0) specifies no motion. Additionally, speed increases or decreases proportionally as the control command deviates from (0, 0).

The speed of left and right wheels (L, R) is represented by normalized values (from −1 to 1). It takes positive values if the wheels rotate forwards and negative values if the wheels turn towards the rear. A joystick is a combination of dual potentiometer [16]. Any movement provides analog voltages. A voltage variation in the *x*-axis provides a displacement of the wheelchair to the right or left, and a variation in the *y*-axis provides the speed variation and forward or back movements. For simplicity, in the rest of the paper, the control command is represented in a polar coordinate system, with two numbers, *ρ* and *θ*, where *ρ* is the distance of the handle to the central position of the joystick and *θ* is the angle with the reference axis.

In the design of the proposed intelligent joystick, any position change provides an analog proportional voltage. The variation in tension on the *x*-axis allows the wheelchair to move to the right or left, and a variation on the *y*-axis allows the wheelchair to vary speed, that is, to move forward or backward.  Table 1 shows the voltage ranges indicated by the joystick.

The relationship between the output of the controller and the voltage given to the motors of the EWC are illustrated by

(1) $$\begin{array}{c} \left(\begin{array}{c} Output1\\ Output2 \end{array}\right)=1.4\times \rho \left(\begin{array}{c} \cos\theta \\ \sin\theta \end{array}\right)+2.5. \end{array}$$

## 6. The Smart Joystick

For each control system, a calibration algorithm is always required as the first task to be performed. The calibration process is necessary because it is difficult to perfectly align the coordinates of a hand with the steering system behind it. Thereby, a calibration algorithm was developed after identifying the sources of joystick movement errors. Several sources of error affect the *ρ* and *θ* coordinates produced by the proposed joystick. The pathological body state of a patient and the noise are the most important sources of error. Any of these errors can produce incorrect data. Hence, it needs to be compensated.

Given the diversity of problems faced by the disabled when driving the wheelchair, we will add to the standard joystick an artificial intelligence algorithm to become a smart controller able to correct these problems and subsequently makes an easier wheelchair.

Deep learning is this branch of machine learning, loosely inspired by how the brain works.

After a thorough study and research, we have chosen an open source library TensorFlow for this application. TensorFlow is a machine-learning library that is used across Google for applying deep learning to many different areas.

In our case, we used this technique, starting by making driving tests for each handicap and deducting eventual causes of driving problems. Then, we put these data (ideal displacement and displacement of handicap) in a recurrent neural network-learning algorithm in order to speculate the optimal one that corrects the different errors appearing during the driving test.

Finally, it is necessary to implant this recurring neural network in the joystick system, which allows each disabled person to have his/her own smart joystick.

### 6.1. Data Collection

**Figure 3:**
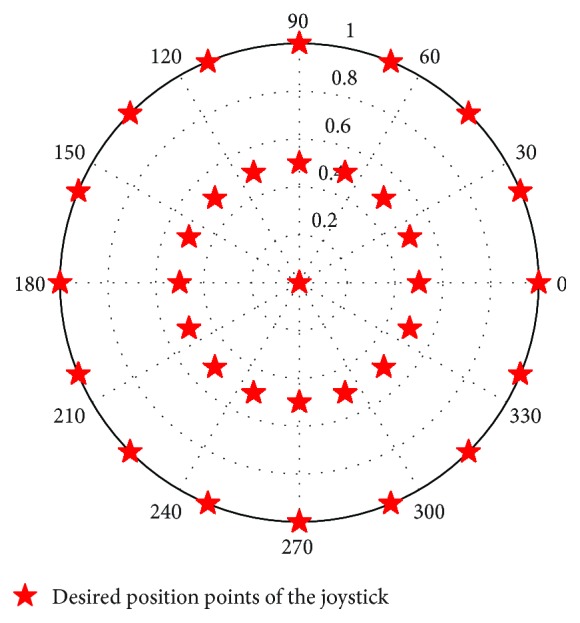
The desired positions of the joystick.

The patient is asked to move his/her joystick in different directions, following a small circle moving on a screen. We have selected 34 points to scan all directions. The points given in Figure 3 represent the desired movement of the map that the patient must follow. The objective of this test is to scan all areas of the joystick. This technique has been well tested in [8] and gives good collection results for samples. To scan all directions, 34 position points were used and each movement lasts 10 seconds. This test is repeated 10 times to collect all possible positions for each desired position. The results obtained are two joystick angle data matrices and amplitude versus time values. These movements will be saved and used as training sets for the recurring neural network.

### 6.2. Neural Network Corrector

The training set of the recurrent neural network is composed of a couple of data which are the desired positions represented by the vector *ρ*_d_ and the corresponding position given by the patient represented by the vector *ρ*, the same for the vector *θ*_d_ and *θ*.

**Figure 4:**
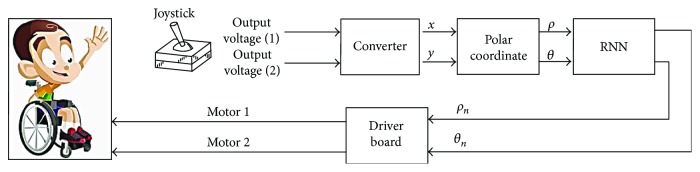
Synoptic of the smart joystick.

The neural network was designed using python 2.7 with Levenberg-Marquardt optimization [17]. Architecture of the neural network used in this study included an input layer of two nodes, the vector(*ρ*_,_*θ*). The number of hidden layers and the number of the nodes are configurable according to the patient and their pathology after several tests with hyperbolic-tangent sigmoid functions. The output layer represents the corrected coordinates. The following figure (Figure 4) illustrates the implementation of the different steps to carry out the proposed joystick used in our experiments.

**Figure 5:**
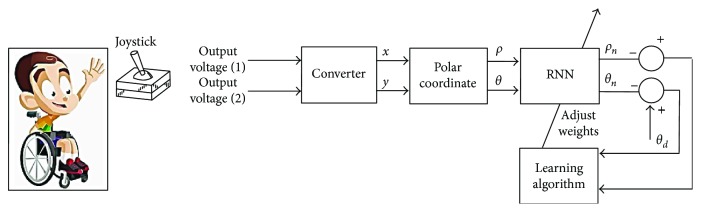
Learning phase of the intelligent joystick.

In the learning step, the neural network training set consists of some data which are the desired positions represented by the vector (*ρ*_d,_*θ*_d_) and the corresponding positions given by the user are represented by the vector (*ρ*_,_*θ*) (Figure 5).

The RNN training model is illustrated in Figure 5. It has many layers of information beginning with an input layer. In this layer, normalized characteristic data is transferred to the model.

The output layer consists of two nodes that provide predicted and corrected data (*ρ*_n,_*θ*_n_). Only one hidden layer was used. The weights are adjusted with a hyperbolic-tangent transfer function. All layers have a bias. The training is given by minimizing the mean square error (MSE).

A supervised feed-forward algorithm was used. In addition, the number of hidden layers and the number of nodes in each of these layers are selected according to the cross-validation method to select the optimal RNN structure.

The training set is divided into five data sets of equal size (fivefold). Then, five training and validation iterations are done. Each iteration has fourfold for training and onefold for validation. In addition, five cross-validation experiments are performed to select the optimal number of layers and nodes in each layer. Finally, the course is completed when the error is less than or equal to a fixed error (MSE).

Evaluation results are produced with a new data set (called a test set). In which the user is asked to follow the movement of a small circle that appears on the screen in positions other than the collection step.

### 6.3. Materials

In order to test our new smart virtual joystick without endangering the patient, we have used a 3D simulator system. With this simulator, a virtual world can be created where a user can drive an electric wheelchair with behavior similar to the real prototype. The virtual world was developed using Unity 3D. It provides a toolkit for creating a 3D simulator with real parameters. The wheelchair control mapping is very important to move in the right direction. The proposed hand controller will be integrated into a Raspberry pi2 in a real EWC. This specific one has already been used for different researches in our laboratory and we still have work to integrate new system controls and functionalities. It is basically composed of 4 wheels, a battery, a joystick, a controller chip, and tow motors. Each motor controls one of the rear wheels.

The proposed controller will be implemented in a real EWC with the parameters indicated in Table 2. This has already been used by various research projects developed in our laboratory [18, 19].

### 6.4. Participants

The aim of this research is to develop a comprehensive method of controlling a wheelchair that ensures safety by using the least possible sensor resources and computing power. We focus on the development of an intelligent human-machine interface, adaptable to the difficulties encountered by the user when using the electric wheelchair. The therapist must systematically precede either by progressive steps or by regressive phases, in which certain parameters must be taken into account, among them, the functional evolution of the gesture and the tremors of the hand.

In our case, among the patient selection criteria, we selected the following criteria for our evaluation:

- Inclusion criteria. Patients will be included if the following criteria are satisfied:
  - Men or women
  - Different patient ages (over 8 years)
- Exclusion criteria. Patients will not be considered if there is at least one of the following criteria:
  - Pregnant women
  - Persons deprived of their liberty
  - Inclusion in another research protocol
  - Voluntary withdrawal by the patient
  - The trial will be discontinued by explicit decision of the doctor

In this project, we are interested in the functional evolution of the movements. After validation of a clinical protocol (reference 06.06.2015) by the University of Tunis and the Mohamed Kassab National Institute Orthopedic, Physical and Functional Rehabilitation Hospital of Tunis, various experimental experiments were launched. The following table (Table 3) illustrates the characteristics of participants in the field trial.

The GFMSS (Gross Motor Function Classification System) [20] functional level can be assessed on the basis of five functional levels (I, minor to V, major difficulties) for children and youth with cerebral palsy. It is useful because it provides clinicians with a clear description of the patient's current motor function. It also provides an idea of what equipment or mobility aids a child may need in the future, such as crutches, walking frames, or wheelchairs.

Level V means that children have physical disabilities that limit voluntary control of movement and the ability to maintain head position. The patient cannot sit or stand alone, even with proper equipment, and cannot walk independently, even if assisted mobility can be used.

FIM [21] measures the patient's dependence in terms of motor, cognitive, psychological, and behavioural abilities by assessing limitations and needs for assistance. It has an ordinal grid of 18 elements, including communication and social cognition.

**Figure 6:**
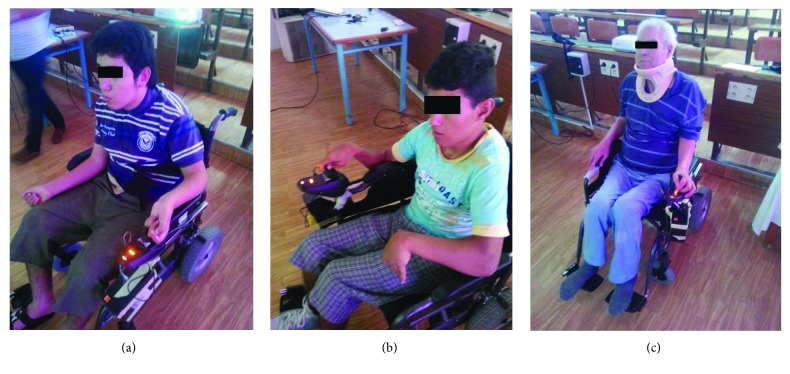
Real participants in the trial phase.

Figure 6 shows the patients who passed the real-time test of the intelligent wheelchair. They have various types of disabilities.

## 7. Experimental Results

**Figure 7:**
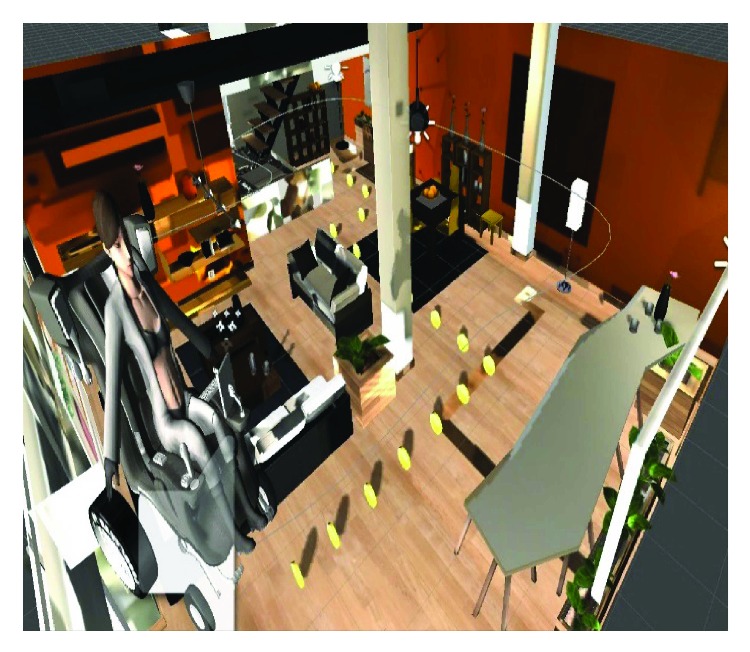
The training trajectory is divided into three parts: driving in a straight line, right turn, and left turn.

The experiments used the virtual simulator (Figure 7) for testing the users' ability in driving the wheelchair with the standard joystick and the proposed joystick. A circuit as defined in the simulator and some objects to be collected (passing through them) were put along the way. These objects (18 coins) determine the trajectory that the wheelchair should follow. The path is shown in the figure below, in which, the user is confronted with some turns (90°) and narrow passages from 100 cm to 150 cm wide. The manoeuvers imposed are therefore delicate and require special attention in order to avoid collisions. This course allows analyzing the behavior of the human-machine system in a very constrained and very congested environment, a frequently encountered situation in indoor environments (apartment, etc.).

During the experimental phase, several measures were taken to determine human capacity in relation to the techniques used. Some indicators have been proposed to analyze the performance of smart wheelchairs [22–24]. These indicators are as follows:

- The movement signals of the joystick
- Travel time (*T*): the time required to complete the mission
- The trajectory of the geometric centre of gravity of the electric wheelchair
- The number of coins (NR) (reference) points to cross (the total number of coins is 18)
- Number of collisions (NC): number of collisions during the mission
- The average time of collision (TC) that corresponds to the time taken by the user in a collision with an obstacle
- Average speed (*V*): average speed during movement.

These data are not independent. For this reason, the increase in the number of collisions will increase travel time by adding a few maneuvers to overcome the situation.

### 7.1. Joystick Calibration

The figure below shows the data given by the first patient during the data collection phase. They, respectively, represent the displacements in polar coordinates (blue colour) and the desired signal (red colour). The superposition of the signals shows the hand movement limitations of this patient in any direction when compared to the referenced signal. After collecting the patient's control signals, we will correct the gaps appearing between signals and the reference one through the proposed RNN algorithm. To do this, we have trained the RNN with the data recorded by the first patient and the desired data until minimizing the mean square error.

The overview of the parameters and their values for each patient are given in Table 4.

**Figure 8:**
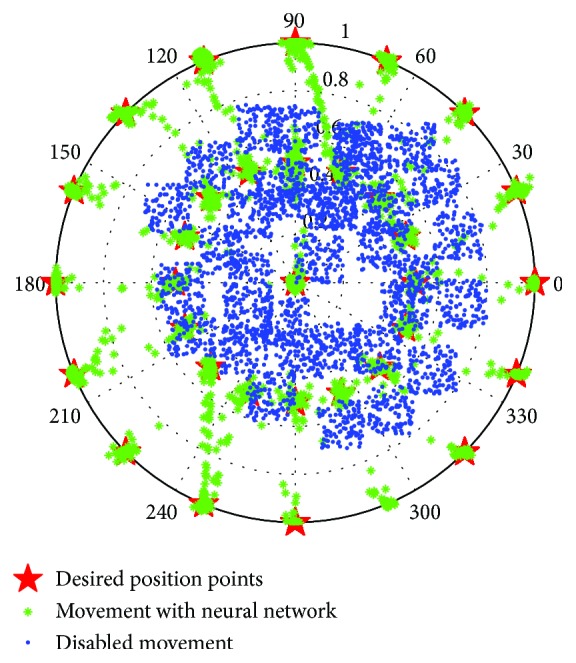
Data recorded during the data collection phase of the first patient and with recurrent neural network corrector in the polar base.

The evaluation results of the RNN are performed with a new set of data (referred as the test set), in which the patient is asked to track the movement of a blue circle that appears on the screen in other positions than the tracking phase.  Figure 8 illustrates these results.

### 7.2. Trajectory Analysis

**Figure 9:**
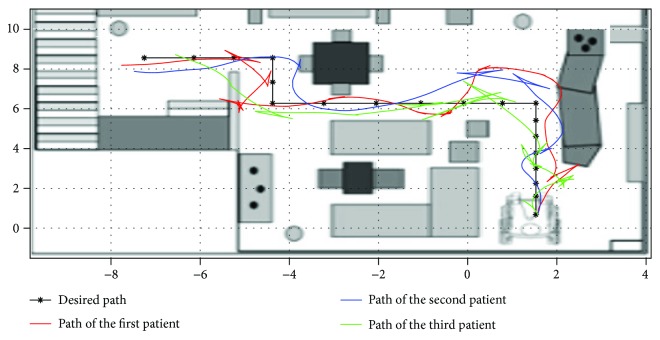
Plots of trajectories as driven by patients with standard driving mode within the scope of experimental test runs.

**Figure 10:**
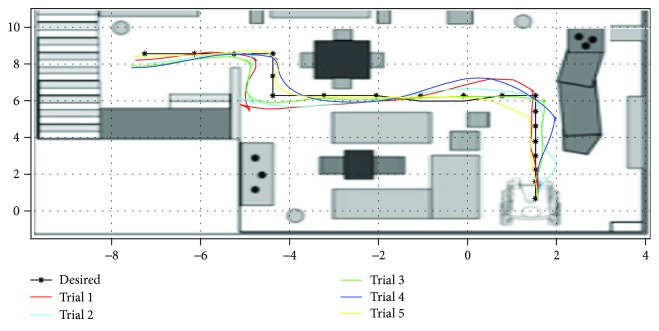
Plots of trajectories as driven by first test patient with assisted driving mode within the scope of experimental test runs.

**Figure 11:**
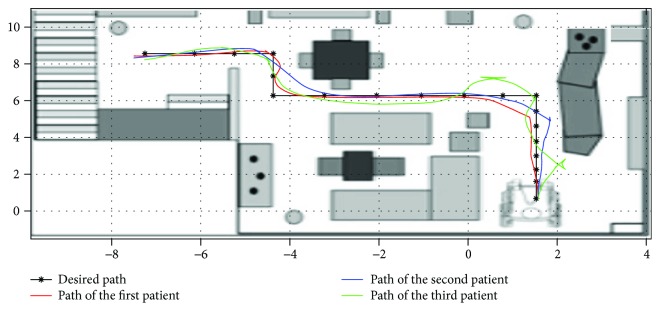
Plots of trajectories as driven by test patients with assisted driving mode within the scope of experimental test runs.

After the creation of the intelligent joystick. We will test its performance against the standard. To do this, we ask a professional and a patient to follow a reference trajectory that appears five times on the 3D prototype. The trajectories followed are shown in the (Figures 9–11).

**Figure 12:**
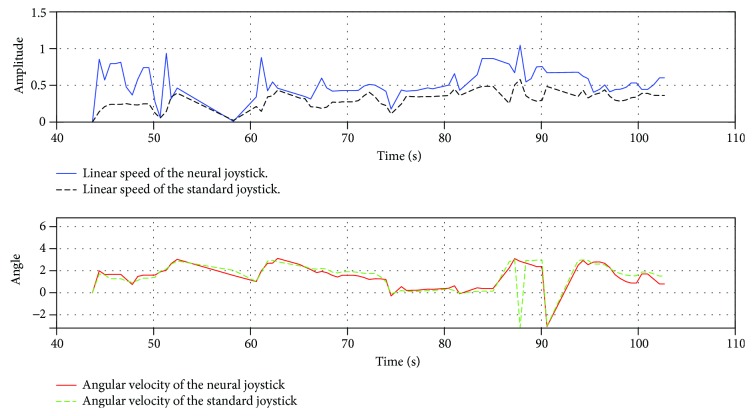
Recorded data from the first patient during the manoeuvre in test number 5.

Figure 12 presents the speed change of the patient during the manoeuvre. It is clear that the correction algorithm of abnormal movements is very relevant for this patient. Equally, it is noted that adding intelligent corrector increases these amplitudes. This leads us to conclude that the proposed neural corrector significantly reduces the time required to complete the navigation task.

The Hausdorff distance calculation (HD) presented in Table 5 shows the Euclidean distance between the patient's trajectory and the desired path. This algorithm makes it possible to estimate the geometric similarity of trajectories [25]. They return a nonnegative number (a distance). Two trajectories are similar if their distance is zero, and the opposite indicates dissimilarity.

The behavioral indicators are mentioned in Table 5, where it can be concluded that all three patients experienced driving difficulties and that these difficulties are fairly clear in the number of collisions measured. We also note that patients are becoming increasingly tired, which results in a loss of time, regardless of how long they travel.

The table also shows an improvement after commissioning the intelligent joystick. This improvement is related to the number of patient transit points, travel time, distance travelled by patients, and the number of collisions.

These results highlight the effectiveness and the reliability of our proposed system, which guarantees a safe navigation for the disabled patient using this EWC.

In this simulation, a clear improvement of the performances in our new assisted navigation mode compared to the standard model is proved. This comparison is done based on the measures of efficiency. The average speed and the number of collisions show that the new strategy generally has a positive effect on control. As a result, execution times are considerably reduced. In addition, the patient acts with the correct amplitude, in addition to the steering control, and there is less variation in the control signal on the wheelchair. Another indicator, such as Hausdorff distance from trajectories, shows large differences in control behavior when using the proposed technique. Note that the effectiveness of the intelligent joystick developed for each patient is confirmed by the doctor during the test phase.

In this context, we performed a 3D simulator manipulated by the intelligent joystick to drive the electric wheelchair with real parameters. The construction of a real electric wheelchair with an intelligent joystick has become possible and easy to implement thanks to the high technological performance.

**Figure 13:**
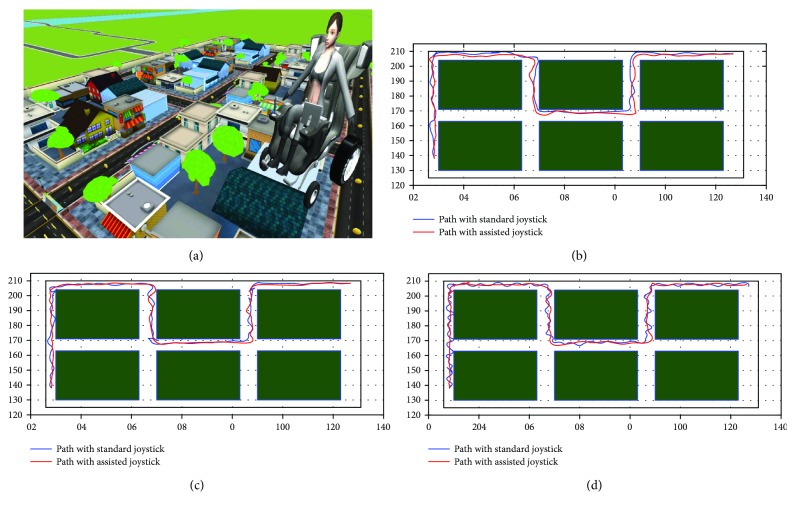
Comparison between the trajectories of the second patient with and without the proposed intelligent joystick: (a) Virtual environment. (b) Trajectories of the first user. (c) Trajectories of the second user. (d) Trajectories of the third user.

To justify our concept, it was tested on the same wheelchair and with the same neuronal structure, another 3D scenario more difficult than the first one where the first scenario presented the navigation in a covered environment and the second one outside represented on Figure 13. The results of these experiments confirm the robustness and efficiency of the proposed intelligent interface.

To validate the intelligent joystick we proposed, we did a real driving test on the electric wheelchair. In practice, once the patient is decided in the virtual simulation to be able to drive his wheelchair safely, we will check this result by the actual driving. This approach is approved by a medical and technical-medical team of the Mohamed Kassab National Institute Orthopedic, Physical and Functional Rehabilitation Hospital of Tunis.

**Figure 14:**
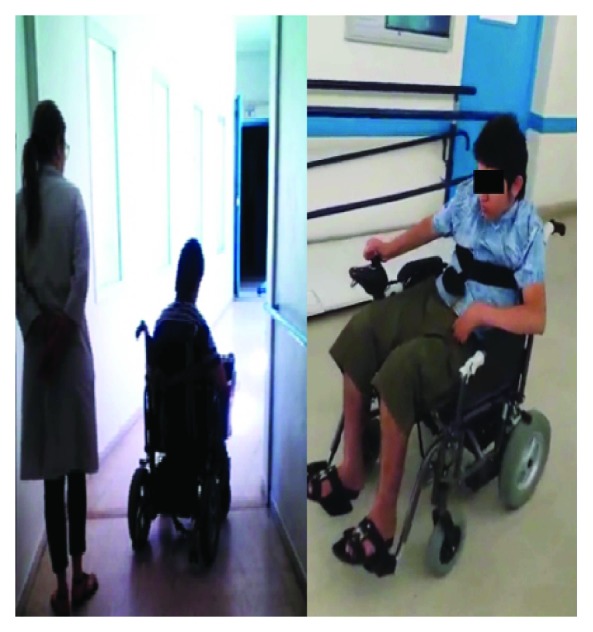
Intelligent wheelchair controls in real environments.

During the experimentation, each patient must drive the electric wheelchair with his or her own smart joystick using the following steps: first of all, the patient performs real tests in open spaces at a reduced speed. It is then allowed to test at higher speeds. Then, the driving tests will take place in the presence of static and dynamic obstacles. Finally, the patient will do his daily activities without modifying the environmental conditions. This includes the movement of people around the wheelchair and many circumstances with secured space to operate, such as doors or narrow passages around equipment or people (see Figure 14). Note that the transition of the patient from one test driving step to another is only made after validation of medical and technical team using a statistical study.

The data of the navigation performance metrics are recorded in Table 6.

After completing the trials with the new joystick, participants were asked to rate their level of satisfaction. To do this, we used the System Usability Scale (S. U. S.). The S.U.S. described in [26] is a scale of ten questions to validate usability tests. It is commonly used in several scientific research projects [27–30].

The three participants were very satisfied with the intelligent joystick offered. They responded with an average satisfaction rate of 92%, 95%, and 84%. In interviews, all patients agreed that they had less physical damage and less effort to navigate with our smart joystick.

## 8. Compared with Other Interfaces

So far, many control methods for wheelchairs have been developed and then they can be classified as intrusive and nonintrusive. They are presented in Table 7. Intrusive methods use electrodes, glasses, a headband, or cap with infrared/ultrasound emitters to measure the user's intention based on changes in the ultrasound waves or infrared reflect [31–33].

On the other hand, nonintrusive methods do not require any additional devices attached to user's body. As shown in Table 7, voice-based and vision-based methods are the basic nonintrusive methods. Voice control is a natural and friendly access method; however, the existence of other noises in a real environment can lead to command recognition failure, resulting in safety problems [34–36]. Consequently, a lot of research has been focused on vision-based interfaces, where control is obtained by recognizing the user's gestures by processing the images or videos obtained via a simple or special camera. With such interfaces, face or head movements are most widely used to convey the user's intentions. When a user wishes to move in a certain direction, it is a natural action to look in that direction, this movement is initiated based on nodding the head, while turning is generated by the head direction. However, such systems have a major drawback, as they are unable to discriminate between intentional behaviour and unintentional behaviour. For example, it is natural for a user to look at an obstacle as it gets close; however, the system will turn and go towards that obstacle [37]. In addition to that, these controls are too tedious for the patient when he has to drive for a long time. Indeed, the patient should, for example, blink his eye or make facial gestures or turn his head across all his path.

In another hand, most of these interfaces mentioned above have predetermined commands to move the wheelchair. It allows only four different commands (forward, backward, left, and right) at a predefined speed, which sometimes presents an annoying limitation for the user.

In our case, the proposed control system is based on nonintrusive methods. It does not require sensors or contraptions attached to the user's body or special human-machine interface on the wheelchair. It offers also a variable speed control in all directions and thereby gives the same benefits as the classical electric wheelchair, known as being the most used until now.

## 9. Conclusion and Perspectives

Nonlinear problems with uncertainties are quite complex. However, with artificial intelligence means (AI), we can create intelligent systems to deal with those uncertainties. Researchers revealed that the RNN is one of the most efficient methods to imitate the human behaviour and to make decisions based on human strategies.

Based on AI methods, we created an intelligent control system and integrated it into an EWC to ensure the safety of its disabled users. This system not only corrects the hand movements but also gives confidence to patients. Therefore, we achieve our goals with the designed system. It helped us to reach the desired movements.

As the present field of research is extremely rich, we can widely develop our work and think about many other possible perspectives such as adding other security requirements like an intelligent control able to pass through open doors; in addition, we can accomplish real-time object detection and adjust the input speed values in order to avoid collisions while moving or even detect the user emotions and find their influence in the navigation state.

## Figures and Tables

**Table 1:** Intelligent wheelchair tension ranges.

	Output1	Output2
Stop	2.5 V	2.5 V
Forward	2.5 V	2.5 V~3.9 V
Backward	2.5 V	1.1 V~2.5 V
Turn right	2.5 V~3.9 V	2.5 V
Turn left	1.1 V~2.5 V	2.5 V

**Table 2:** Characteristics of the proposed intelligent wheelchair.

Wheelchair parameters		A processing device (Raspberry Pi II model B)	
Height	89 cm	Price	$39.99
Width	61 cm	Chip	Broadcom BCM2836
Frame weight with batteries	58 kg	Processor	ARMv7 quad-core
Load capacity	110 kg	Processor speed	900 MHz
Linear velocity	8 km/h	Voltage and power draw	650 mA @ 5 V
Ø front wheels	20 cm	GPU`	Dual Core VideoCore IV Multimedia Co-Processor
Ø rear wheels	30 cm	Size	85 × 56 mm
Stopping distance	1 m	Memory	1 GB SD RAM @ 400 MHz
Noise	65 dBA	GPIO	40
Battery life	20 km	USB 2.0	4
Battery	2 × 12 v 28 Ah	Ethernet	10/100mb Ethernet RJ45 Jack
Engine power	2 × 220 W 24 V	Audio	Multichannel HD audio over HDMI, analog stereo from 3.5 mm headphone jack

**Table 3:** Patient characteristics.

Characteristics	Patient I	Patient II	Patient III
Sex, age, mass (kg)	Male, 15, 54	Male, 16, 45	Male, 66, 75
Diagnosis	Posttraumatic tetraplegia	Cerebral palsy	Cervical myelopathy
Motor disability and clinical symptoms	Spastic tetraplegia C5	Dyskinetic tetraplegia	Spastic tetraplegia
Handedness	Left-handed	Right-handed	Left-handed
Functional level	FIM: 82	GMFCS: V FIM: 89	FIM: 86

**Table 4:** Structure and training results for the neural network models.

Patient	Net structure	Training function	Momentum	MSE test	Iterations	Time
Patient I	2-16-16-2	Hyperbolic-tangent sigmoid	0.5	0.00816	1437	0:05:51
Patient II	2-14-14-2	Hyperbolic-tangent sigmoid	0.5	0.00898	1511	0:05:17
Patient III	2-17-17-2	Hyperbolic-tangent sigmoid	0.5	0.009238	1848	0:08:53

**Table 5:** Performance indices from the users' paths.

	Path length (m)	*T* (sec)	*V* (m/s)	NC	TC (s)	NR	HD
Path made by an expert	15.8983	71.39407	0.2227	0	0	18	0.6737
Disabled path (patient I)	38.0598	249.3485	0.1526	5	5.8832	11	1.4203
Intelligent joystick path (patient I)	18.1164	102.6936	0.1764	0	0	15	0.9215
Disabled path (patient II)	25.2091	197.7362	0.1275	3	4.5483	8	1.6728
Intelligent joystick path (patient II)	16.4844	111.8486	0.1474	0	0	14	0.8633
Disabled path (patient III)	36.2845	225.3885	0.1610	5	5.9554	8	1.9099
Intelligent joystick path (patient III)	21.1313	128.2292	0.1648	1	2.6652	13	1.0678

**Table 6:** Performance indices for assessing simulated a wheelchair driven by the proposed joystick.

	Collisions	Incomplete assignment	Path length (m)	Time (s)	Mean velocity (m/s)	Static time (s)	Total time (s)
Expert	0	0	178	143	1.24	0	143
Patient I (PI)	0	0	183.1	158	1.158	9	167
Patient II (PII)	0	0	183.8	159	1.155	7	166
Patient III (PIII)	1	1	186	169	1.100	14	183

**Table 7:** Wheelchair controls in literature.

		Wheelchair	Feature	Device	Commands
Intrusive interfaces		Chen et al. [31]	Head orientation	Tilt sensors, microprocessor	Go, back, left, and right
		SIAMO project [32]	Eye gaze	Electrode	Go, back, left, and right
		Wheelesley [33]	Eye gaze	Electrodes (EOG)	Go, back, left, and right

Nonintrusive interfaces	Voice	SIAMO project [32]	Voice	Microphone	Go, back, left, and right
		ROB Chair [34]	Voice	Head microphone	Go, stop, speed up, speed down, and rotate
		NAVChair [35]	Voice	Computer	Go, stop, back, left, and right
		TAO project [36]	Voice	Microphone	Go, stop, back, left, right, and speed down
	Vision	Yoshida et al. [38]	Face	Tow video camera	Go, stop, left, and right
		HGI [37]	Head & nose	Webcam, data acquisition board	Go, left, right, speed up, and speed down
		SIAMO [32]	Head	CCD camera	Go, left, right, speed up, and speed Down
		Rabhi et al. [8]	Hand	Webcam, data acquisition board	Analog commands
	Proposed smart joystick		Joystick	Data acquisition board	Analog commands
